# A yeast model of 5‐oxoproline accumulation reveals a general toleration to 5‐oxoproline

**DOI:** 10.1002/2211-5463.70254

**Published:** 2026-07-15

**Authors:** Pratiksha Dubey, Vaishnavi Sanjayan, Praveen Singh, Shantanu Sengupta, Anand Kumar Bachhawat

**Affiliations:** ^1^ Indian Institute of Science Education and Research Mohali Punjab India; ^2^ Saint Louis University School of Medicine St. Louis MO USA; ^3^ CSIR‐Institute of Genomics & Integrative Biology (IGIB) Delhi India; ^4^ Buck Institute for Research on Aging Novato CA USA

**Keywords:** 5‐oxoproline (5‐OP), glutathione metabolism, pyroglutamic acid, redox, transcriptomics, yeast model

## Abstract

5‐oxoproline (5‐OP) or pyroglutamic acid is an intermediate in the degradation arc of the glutathione cycle. It is metabolized into glutamate through the action of the 5‐oxoprolinase enzyme, the only enzyme known to act on this metabolite. 5‐OP has long been known to be relatively inert with a proposed role as an osomoprotectant. Recent studies on the 5‐oxoprolinase enzyme in mammalian cells have, however, shown that knockdown or deletion of 5‐oxoprolinase makes mice (and humans) prone to heart failure, an effect ascribed to oxidative stress caused by a twofold increase in 5‐OP. To examine the consequences of 5‐oxoproline accumulation more rigorously, we created a yeast model for 5‐oxoproline accumulation. Using this model, we observed retardation of growth only when intracellular levels of 5‐OP were increased 12‐ to 20‐fold over normal levels. Performing an analysis of transcriptomic changes under these conditions, we observed a large number of genes were differentially regulated and while there was no unifying dysregulated pathway, there was an upregulation of various efflux pumps. Ultimately, modulating the expression of these genes by knockout or overexpression highlighted that many of the upregulated genes were involved in the cellular response to 5‐OP accumulation. However, our results failed to show any significant oxidative stress response. In conclusion, our study suggests a need to reevaluate previous suppositions of the 5‐OP induced oxidative stress response and proposes alternate mechanisms for this effect.

## Graphical abstract

Using a yeast model, we show that even high accumulation of 5‐oxoproline causes only mild cellular stress and does not trigger oxidative stress. Instead, cells adapt by activating efflux pumps and diverse protective pathways, suggesting that previously proposed harmful effects of 5‐oxoproline may arise from indirect metabolic imbalances rather than the molecule itself. *Created in BioRender. bs, V. (2026) https://BioRender.com/492ypa9.

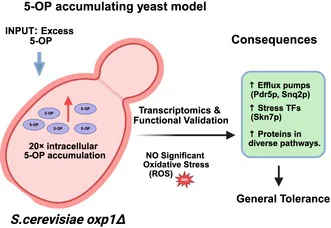

**Abbreviations**
5‐OP: 5‐oxoproline
ABC transporter: ATP‐Binding Cassette transporter
CD147: Basigin (MCT1 chaperone protein)
Chac1: ChaC glutathione‐specific γ‐glutamylcyclotransferase 1
GO: gene ontology
GSH: glutathione
LC–MS: Liquid Chromatography–Mass Spectrometry
MAPK: Mitogen‐Activated Protein Kinase
MCT1: Monocarboxylate Transporter 1
MOP family: Multidrug/Oligosaccharidyl‐lipid/Polysaccharide exporter family
MRM: Multiple Reaction Monitoring
OPLAH: 5‐oxoprolinase (mammalian enzyme)
OXP1: 5‐oxoprolinase gene in yeast
qRT‐PCR: Quantitative Reverse Transcription PCR
RNA‐seq: RNA sequencing
ROS: Reactive Oxygen Species
SD medium: synthetic defined medium
YNB: Yeast Nitrogen Base

5‐oxoproline (5‐OP), also known as pyroglutamic acid, is widespread in nature [1]. It is an intermediate of the glutathione cycle, a cycle that describes glutathione metabolism in living cells [2]. 5‐oxoproline can also be found at the N terminus of some proteins, which is formed from the spontaneous cyclization of N‐terminal glutamate or glutamine in proteins [3, 4]. At the N‐terminal sites of proteins, 5‐oxoproline contributes to both function and stability [5]. However, as a free metabolite, its functions are less clear. It appears to have a role in glutamate storage, and it has also been suggested to function as an osmoprotectant in microbes [1, 6, 7] and a moisturizer in mammals [8, 9].

The only enzyme that acts on 5‐oxoproline is 5‐oxoprolinase. This enzyme hydrolyses 5‐OP to yield glutamate. The enzyme is considered to be one of the most sluggish enzymes [10]. Thus, 5‐oxoproline in cells often accumulates, and some of it is found to be secreted into the plasma [11]. Recent studies with both humans and mice have revealed that cardiac injury leads to 5‐oxoprolinase depletion, resulting in elevated 5‐OP levels in the plasma [12, 13]. The study also found that cardiac function after ischemic injury could be rescued by overexpressing 5‐oxoprolinase [12, 13]. The study concluded that 5‐OP accumulation was causing oxidative stress. This was surprising for two reasons. First, 5‐OP appears to be a relatively inert molecule, and its previous role has in fact been suggested in osmoprotection. Second, the increased levels in plasma observed during 5‐oxoprolinase knockdown was only twofold [12, 13]. This was not a very high increase in plasma level 5‐OP since 5‐OP levels are normally observed in plasma, and levels are also known to vary with diet [14].

It was important to understand therefore, if it was 5‐oxoproline accumulation *per se* that might be important, or some other underlying mechanism (such as decreased intracellular glutathione). We have accordingly begun to investigate more carefully whether 5‐OP accumulation in cells does indeed cause oxidative stress. To address this question, we have created a yeast model for 5‐OP accumulation. We initially tried creating a model where 5‐OP would be generated endogenously. However, it could not lead to significant elevations in 5‐OP. We finally established a model where a yeast strain lacking 5‐oxoprolinase (*oxp1Δ* strain) was treated with excess 5‐OP in the medium. Due to deletion of the 5‐oxoprolinase gene, 5‐OP cannot be metabolized, leading to 5‐OP accumulation. After establishing a model that could cause between 12‐ and 20‐fold increase in 5‐OP levels, the consequences of 5‐OP accumulation were investigated using transcriptomics. RNA‐seq analysis followed by GO analysis revealed that xenobiotic export and efflux pathways were upregulated. In addition, transcription factors in diverse pathways were mildly upregulated including the stress response factor Skn7p; several different enzymes and membrane proteins were also upregulated. We confirmed using a deletion strain that the knockout of many of these different genes, though not all, did in fact cause them to be more sensitive to 5‐OP. Our study suggests that 5‐OP accumulation to 12‐fold higher levels causes only a mild stress to the cell through multiple pathways, which cumulatively leads to the growth defect in *oxp1Δ* strain.

## Materials and methods

### Chemicals and reagents

All chemicals used were of analytical reagent grade. Media components were purchased from Hi Media (India), Merck (Germany), and BD Difco (USA). Oligonucleotides were purchased from Merck (Germany) and GCC (India). Restriction enzymes, Phusion polymerase, dNTPs, and other modifying enzymes were obtained from New England Biolabs (Beverly, MA, USA). Gel‐extraction kits and plasmid miniprep columns were obtained from QIAGEN (Germany) or Thermo‐Fischer Scientific (USA). 5‐oxoproline and glutathione were purchased from Merck (Germany).

### Strains, media and growth

The *Escherichia coli* strain DH5α was used as a cloning host. The list of yeast strains used in this study is shown in Table S1. Yeasts were routinely maintained on YPD medium. The minimal medium consisted of yeast nitrogen base (YNB), glucose, and ammonium sulfate, and was supplemented with the required amino acids. The medium was further supplemented with varying concentrations of 5‐oxoproline. Yeast DNA Isolation and Yeast Transformation—Yeast chromosomal DNA was isolated by the glass bead lysis method and yeast transformations were carried out using the lithium acetate method [15].

### Growth studies

The growth assay for the endogenous model for 5‐OP accumulation was done with *met15Δdug3–2ecm38Δoxp1Δ* strains transformed with human Chac1 along with OXP1 (test) and human Chac1 with empty vector (control). The transformants were grown overnight on synthetic medium (without ura and his). Further, the inoculum (25 mL) was initiated at 0.01 OD_600_ on complete synthetic medium (without ura, his and met) supplemented with 100 μM glutathione (GSH) and growth was monitored. Cells were harvested at mid logarithmic phase and 5‐OP quantification was done using mass spec.

For exogenous model, the *oxp1Δ* cells and *oxp1Δ* cells transformed with MCT‐1 and CD‐147 were grown in complete synthetic medium overnight; WT cells (with or without empty vector) were grown on same condition as a control. Further, the inoculum (25 mL) was initiated at 0.01 OD_600_ on complete synthetic medium supplemented with different concentrations (1 mm, 2.5 mm, 5 mm, and 10 mm) 5‐oxoproline and growth was monitored for 32 h. A without treatment condition was taken as a control.

### Growth assay by dilution spotting

For growth assay, the different strains were grown overnight in minimal ammonia medium without Uracil/Leucine/histidine and reinoculated in fresh medium to an OD600 of 0.1 without Uracil/Leucine/histidine depending upon the selection marker and grown for 6 h. The exponential‐phase cells were harvested, washed with water, and resuspended in water to an OD600 of 0.2. These were serially diluted to 1:10, 1:100, and 1:1000. Of these cell resuspensions, 10 μL was spotted on minimal medium containing 50 mm or 100 mm 5‐oxoproline. The plates were incubated at 30 degrees for 3–6 days and photographs were taken.

### RNA sequencing

To study the consequences of 5‐OP accumulation, AB6302 strain was grown in SD medium containing 5 mm 5‐oxoproline (test) or 0 mm 5‐oxoproline (control). The mid log phase cells were harvested, washed with water, and frozen. Total RNA was isolated using Qiagen RNeasy mini kit (Cat # 74106), RNA sequencing libraries were prepared with Illumina‐compatible NEBNext® Ultra™ II Directional RNA Library Prep Kit (New England BioLabs, MA, USA) and were carried out by Genotypic Pvt.Ltd. Further, paired‐end Illumina Next‐Generation Sequencing was performed at Genotypic Technology Pvt. Ltd., Bangalore, India. The experiment was done in duplicates, and the data were deposited in NCBI (Accession no. PRJNA1345586).

### GO enrichment analysis

GO enrichment analysis was carried out using web‐based ShinyGO version 0.85 ([16]). The list of genes either upregulated or downregulated in response to 5‐oxoproline were subjected to GO enrichment analysis. Top biological processes selected using the *e* value < 0.05 and fold enrichment >10.

### Gene cloning

*KDX1*, *YHR033W*, *OSI1*, *ERC1*, and *FEX2* genes were cloned in pRS416TEF by PCR amplification followed by homologous recombination in yeast as described previously [17]. The gene‐specific primers carried overlapping sequences for pRS416TEF, and an internal restriction site BamHI and XhoI were also added in forward and reverse primers respectively (Table S2). Two further sets of primers were also designed to amplify the vector in two different fragments carrying the overlapping sequences for homologous recombination. One fragment of the vector called CEN fragment, as it contains CEN; another called URA fragment, as it contains URA marker. For homologous recombination, the CEN and URA fragments, along with the SEO1 gene fragment carrying vector overlapping sequences, were transformed in yeast using the lithium acetate method and plated on SD‐URA plates. A few colonies were picked up, and desired recombinants were confirmed by PCR. The plasmids were isolated after passage through E. coli, and digestion by the restriction enzymes BamHI and XhoI confirmed the presence of the clone. Human MCT‐1 and CD‐147 cDNA were purchased from Sino biologicals, cloned in pRS416‐TEF and pRS315‐TEF using restriction digestion method under EcoRI and XhoI sites, respectively. Other clones used in this study were previously available in the lab.

### Metabolite extraction

Intracellular metabolites for MS‐based targeted metabolomics were extracted using 80% methanol. Briefly, the yeast cell pellet was collected by centrifugation in a microcentrifuge tube and washed three times with sterile water, then quenched with prechilled 80% methanol (kept at −80 °C), followed by 7 cycles of bead beating (using glass beads). The solution was centrifuged at 15 000 ***g*** at 4 °C for 15 min, and the supernatant was collected in a fresh tube and dried using a vacuum concentrator operated at room temperature. Extracted metabolites were reconstituted in 150 μL of 50% methanol with 0.1% formic acid, centrifuged at 15 000 ***g*** for 10 min, and transferred to HPLC vials.

### Mass spectrometry

The data were acquired using a multiple reaction monitoring (MRM) method on a triple quadrupole hybrid ion trap mass spectrometer (QTRAP 6500+, SCIEX) coupled with an ExionLC UHPLC system (SCIEX). Optimized source and gas parameters were used, and data acquisition was performed through the Analyst 1.6.3 software in positive ion scan mode. 5 μL of metabolites suspension was loaded and resolved on an Acquity UPLC BEH C18 column (1.7 μm, 2.1 × 100 mm, Waters) using mobile phases of water with 0.1% formic acid (buffer A) and acetonitrile with 0.1% formic acid (buffer B) with a flow rate of 0.3 mL/min and 8‐min‐long gradient. The gradient program was employed as follows: Buffer B was raised from an initial 2% to 30% in 2.1 min. In the next 2 min, buffer B was increased to 40% and further to 45% in the next 0.7 min. The buffer B concentration was ramped up to 90% in the next 0.1 min and kept constant for the next 1.1 min. Buffer B concentration was brought to an initial 2% concentration in the next 0.5, and the column was equilibrated at this buffer condition for 1.5 min before the next sample injection. Relative quantification was performed using the MultiQuantTM software v.3.0 (SCIEX). A pooled QC from samples was used to analyze the technical variability.

### q‐RT‐PCR

For genes, the primer set listed in Table S2 was used with a final reaction volume of 5 μL containing using Maxima SYBR Green qPCR Master Mix (Fermentas, USA). PCR conditions were 95 °C for 3 min, then 40 cycles consisting of denaturation at 95 °C for 10 s, annealing at 60 °C for 10 s and extension at 72 °C for 30 s, followed by the melting curve protocol with 10 s at 95 °C and then 60 s each at 0.5 °C increments between 65 °C and 95 °C. The reactions were performed in triplicate for each sample. The relative amounts of target gene expression for each sample were calculated using the Livak method formula 2^−(ΔΔCT)^ against an endogenous control actin for genes. Finally, the fold change against the control gene is calculated and plotted. Data analysis was performed by Graph pad prism using a paired t‐test. The significance of differences between means was calculated at a 5% level (*P* < 0.05).

### ROS measurement using FACS

Overnight yeast cultures of *oxp1Δ* cells were inoculated into fresh complete SD medium supplemented with 5‐oxoproline and incubated at 30 °C until reaching the exponential growth phase. Three replicate cultures were harvested (approximately 3 million cells each) by centrifugation at 6000 g for 10 min. The cell pellets were then washed twice with 1× phosphate buffer (0.1 m, pH 7.4). For a positive control, one set of samples was treated with 1.5 mm of diamide per 1 mL of sample for one hour at 30 °C. Subsequently, all samples, including the diamide‐treated control and test samples, containing approximately 1 million cells each, were incubated with 250 μL of 80 μm H2DCFDA dye prepared in DMSO in the same buffer for 20–30 min at 30 °C in the dark. After incubation, the cells were washed with 1× PBS and resuspended in 500 μL of fresh 1× PBS. Finally, cellular fluorescence intensity, an indicator of reactive oxygen species (ROS) production, was measured using a FACS Aria III Fusion machine under FITC channel.

### *In vitro* ChaC1p‐Dug1p coupled enzymatic assay for inhibition studies

5 ng of recombinantly purified human ChaC1 protein was incubated with 5‐oxoproline (dissolved and diluted in sterilized distilled water) for 30 min in a 50 μL reaction mixture containing 50 mm Tris‐Cl (pH 8.0) and 5 mm DTT. Immediately after, 2 mm of the substrate, glutathione, was added to this mixture and further incubated for 30 min. The enzyme was inactivated by heating at 95 °C for 5 min. To this, 10 μL of reaction mixture containing 5 μg of Dug1p and 20 μm MnCl_2_ was added and incubated at 37 °C for another 1 h. Cysteine liberated from the above reaction was measured using a ninhydrin‐based method [18].

### Statistical analysis

The data have been analyzed using Student's t‐test and one‐way ANOVA with *P* value cut off of 0.05 using Graph Pad Prism.

## Results

### Creation of a yeast model for 5‐oxoproline accumulation

To study the effect of intracellular accumulation of 5‐OP in yeast cells, we needed a system that could result in accumulation of a high amount of intracellular 5‐OP. We initially conceived of two broad approaches. The first approach was to generate high levels of 5‐OP endogenously by transforming into yeast a 5‐OP generating enzyme and the second approach was to exogenously provide different levels of 5‐OP in the medium enabling its accumulations within the cell.

In the first approach, we used heterologously expressed human Chac1. Chac1 degrades glutathione efficiently to generate 5‐OP and Cys‐gly [19]. We introduced this into a strain that was deleted for the endogenous glutathione degradation pathways Ecm38p and Dug3p. Ecm38p is a γ‐glutamyl transpeptidase that cleaves glutathione to yield glutamate and Cys‐gly, and Dug3p is a component of the Dug2p–Dug3p complex that also yields glutamate and Cys‐gly on glutathione degradation [20]. They were both distinct in their activity compared to Chac1, which cleaves glutathione to yield 5‐OP and Cys‐gly. Therefore, we used a strain background that is lacking both these enzymes. We also used the *met15Δ* background. This is an organic sulfur auxotroph, and it enabled us to evaluate whether Chac1 was indeed degrading glutathione in the cells. Since the degraded glutathione would release cysteine to provide the sulfur source required. We also created a 5‐oxoprolinase deletion (*oxp1Δ*) in this background. This strain, *met15Δdug3*–*2ecm38Δoxp1Δ*, was transformed with either the *OXP1‐expressing* clone (TEF‐*OXP1*) or with an empty vector. Those with the TEF‐*OXP1* clone would degrade 5‐OP generated from Chac1, while those transformed with the empty vector were expected to have 5‐OP accumulated. We found that the vector‐transformed strains were showing slightly slower growth as compared to *OXP1* overexpressed strains (Fig. S1A). 5‐OP levels were then estimated in the cell extracts using LC–MS. However, we could not observe any accumulation of 5‐OP in the vector‐transformed cells (Fig. S1B) compared to the *OXP1* transformed cells. In contrast, there was some small increase in 5‐OP levels in the *OXP1*‐transformed cells in contrast to the expectations. This suggests that this model was not really suitable as a model for 5‐OP accumulation.

In the second approach, we examined the use of exogenous 5‐OP to increase 5‐OP levels in the cell. Although in mammalian cells, a 5‐OP transporter has been described (SLC16A1/MCT1) [21], in yeast, the transporter of 5‐OP is not known. We therefore initially evaluated whether heterologous expression of the human 5‐OP transporter, MCT1, would enhance the sensitivity of yeasts to 5‐OP. MCT1 in humans is a pyruvate transporter that was also shown to transport 5‐OP [21]. Its expression in yeast was shown to permit transport of pyruvate in yeast, but the activity to transport 5‐OP was not examined [22]. The surface expression of MCT1 was enhanced with expression of CD‐147, although even then only 3% was surface localized [23]. The ability of the heterologously expressed MCT1 under the TEF promoter to enhance 5‐OP sensitivity in yeast was thus examined (with and without CD147). We observed that in these cells, the sensitivity to 5‐OP was significantly enhanced since even at 1 mm 5‐OP the growth of cells was retarded in these transformants (Fig. S1C). When the cells only contained hMCT1, there was some growth retardation in the presence of 5‐OP, but this was enhanced when MCT1 and CD147 were both expressed together (Fig. S1D,E). We quantified 5‐OP levels and there was a significant accumulation observed even at 1 mm concentration (Fig. S1F). These results indicate firstly that human MCT1 can transport 5‐OP in yeast, and secondly that the growth retardation of yeast seen in the presence of 5‐OP is a consequence of its transport into the cell and not due to its action on the surface membrane. However, rather than using the heterologously expressed proteins which had reportedly limited surface localization, we decided to resort to the yeast model without any heterologously expressed proteins to simplify the subsequent downstream conclusions.

**Fig. 1:**
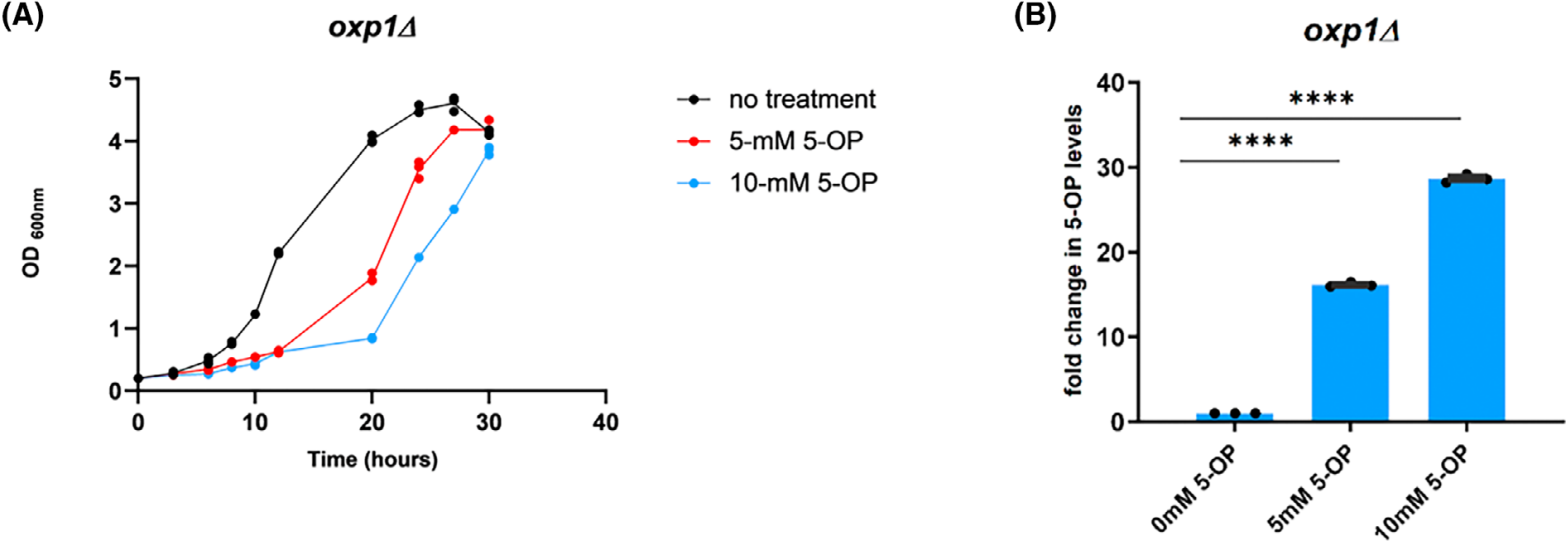
Growth and 5‐oxoproline estimation in *oxp1Δ* model and its consequences: (A) Growth of *oxp1Δ* cells treated with 5 mm or 10 mm 5‐OP. (B) Intracellular 5‐OP levels in *oxp1Δ* cells treated with 5 mm or 10 mm 5‐OP compared to untreated control, quantified by mass spectrometry. All experiments were performed independently three times. Statistical analysis was carried out using one way ANOVA with multiple comparisons. Significance is indicated as (****P < 0.0001). Error bars represent SEM.

We evaluated increasing amounts of 5‐OP in the media to *oxp1Δ* cells and looked for concentrations at which growth defects were seen (Fig. 1A). In liquid medium, we found that beginning with 5 mm, and more significantly at 10 mm, we could see a retardation in growth and we subsequently used these concentrations. However, we did not see such significant growth retardation in wild‐type cells (BY4741) at a 5 mm concentration of 5‐OP (Fig. S1G). We then estimated 5‐OP levels in *oxp1Δ* cells treated with either 5 mm 5‐OP or 10 mm 5‐OP after extensively washing off any surface 5‐OP. We observed that in the 5 mm 5‐OP‐treated cells the intracellular levels of 5‐OP increased 12‐fold, while in 10 mm, it had increased 20‐fold (Fig. 1B). These elevated levels seemed sufficient to evaluate the consequences on the cell.

### Transcriptomic analysis to study the consequences of 5‐OP accumulation

To obtain insights into the consequences of cellular treatment of 5‐OP and its accumulation in yeast, we carried out a transcriptome sequencing of *oxp1Δ* cells exposed to 5 mm 5‐oxoproline vs 0 mm 5‐oxoproline. We chose 5 mm because at this concentration, intracellular 5‐OP levels were significantly elevated (~12 fold) and it was beginning to retard the growth of the cells. The transcriptomic approach was done under these two conditions so that we would obtain an unbiased insight into (a) the possible pathways or proteins that were targets of 5‐OP leading to their inactivation and consequently the effect on growth and (b) the cellular response to 5‐OP toxicity enabling the cells to tackle the 5‐OP treatment and accumulation. For these experiments, cells were grown on minimal medium supplemented with 5 mm 5‐OP (or No 5‐OP) and harvested at log phase of growth followed by RNA isolation and RNA seq analysis using the Illumina platform. We analyzed the data after removing those with insignificant *P* value (>0.05), and carried out a GO enrichment analysis using the ShinyGO algorithm [16]. We observed, while using cutoffs of *E* < 0.05 and Fold enrichment >10, that among the biological processes only the xenobiotic export, xenobiotic detoxification and export across the plasma membrane (Table S3). All these pathways largely included the same subset of genes, although it missed one in the class, *FEX2* that we picked up manually. Among the downregulated genes (Table S4), using the same cutoffs, no biological processes appeared as significant in the ShinyGO analysis. Among the upregulated genes, genes of the oxidative stress pathway seemed particularly absent, instead we found many genes that were twofold induced and manual observations also confirmed that these were in multiple categories or pathways (Table 1), We used a cut off of log_2_fold = 1.20. The upregulated genes include the efflux pumps (*PDR5*, *ERC1*, *SNQ2*, *FEX2*), membrane transporters (*PMA1*, *PNS1*, *YLR413w*, *PRM10*, *WSC2*, *FLC2*), transcription factors (*HCM1*, *SKN7*, *SMP1*, *TBF1*), and enzymes (*KDX1*, *DBP8*, *HMT1*, *AAA1*, *IME2*, *YHR033W*). However, there did not seem any particular pathway that was primarily affected other than the xenobiotic export or efflux pathways.

**Table 1:** List of upregulated genes; transcriptomics analysis was done between *oxp1Δ* (AB6302) cells grown on 5 mm 5‐OP (test) vs 0 mm 5‐OP (control) as seen by RNA seq. AB6302 cells were grown in SD medium containing 5 mm 5‐OP or 0 mm 5‐OP and harvested at mid logarithmic phase. RNA seq analysis was carried out to find out the upregulated genes from different pathways as described in the Methods. Only the genes that had a *P* < 0.05 and log_2_ fold change >1 were included in the list.

*Gene*	Log2foldchange	*P* value	Protein function	Gene ontology
*YHR033W*	1.88	1.82E‐06	Uncharacterized protein	Unknown function
*YLL066C*	1.79	0.009487	Y′ element ATP‐dependent helicase	DNA helicase activity
*YKL161C (KDX1)*	1.68	3.72E‐05	Serine/threonine‐protein kinase	Protein phosphorylation
*YJL108C (PRM10)*	1.66	0.002075	Pheromone‐regulated membrane protein 10	Response to pheromone
*YLR413W (INA1)*	1.54	0.000328	Cell membrane protein	Cell adhesion
*YOR144C (ELG1)*	1.44	0.00318	Telomere length regulation protein	DNA replication
*YFR032C (RRT5)*	1.44	0.170165	Regulator of rDNA transcription protein 5	rDNA transcription regulation
*YPL279C (FEX2)*	1.44	0.017223	Fluoride export protein 2	Fluoride ion transport
*YHR032W (ERC1)*	1.43	0.011175	Ethionine resistance‐conferring protein 1	Response to toxic substance (ethionine)
*YOR153W (PDR5)*	1.41	0.000132	Pleiotropic ABC efflux transporter of multiple drugs	Drug export; ATPase activity
*YGL008C (PMA1)*	1.41	0.058436	Plasma membrane ATPase 1	Proton transport; ATP hydrolysis
*YKL071W (OSI1)*	1.38	0.029716	Uncharacterized oxidoreductase	Oxidoreductase activity; stress response
*YER153C (PET122)*	1.37	0.006165	Mitochondrial protein	Translation activator
*YPL128C (TBF1)*	1.35	8.35E‐05	DNA binding protein	DNA binding; transcription regulation
*YDR501W (PLM2)*	1.34	0.005771	Uncharacterized protein	Unknown function
*YBR182C (SMP1)*	1.34	0.010139	Transcription factor	Transcription regulation; MAPK signaling; stress response
*YOR161C (PNS1)*	1.31	0.025734	pH nine‐sensitive protein 1	Vacuolar acidification
*YCR065W (HCM1)*	1.30	0.000112	Forkhead transcription factor	Transcription regulation
*YHR169W (DBP8)*	1.30	0.003044	ATP‐dependent RNA helicase	rRNA processing
*YJL187C (SWE1)*	1.28	0.000211	Mitosis inhibitor protein kinase	Cell cycle checkpoint
*YHR206W (SKN7)*	1.27	0.014344	Transcription factor	Oxidative stress response
*YBR034C (HMT1)*	1.25	0.002766	Protein arginine N‐methyltransferase 1	Protein methylation
*YNL141W (AAH1)*	1.24	0.000695	Adenine deaminase	Purine metabolism
*YOL084W (PHM7)*	1.23	0.011321	Phosphate metabolism protein 7	Phosphate ion homeostasis
*YNL283C (WSC2)*	1.23	0.004092	Cell wall integrity and stress response component 2	Cell wall organization; stress response signaling
*YAL053W (FLC2)*	1.23	0.000806	Flavin carrier protein 2 (FAD transporter 2)	FAD transmembrane transport
*YOR207C (RET1)*	1.23	0.000607	DNA‐directed RNA polymerase III subunit	Transcription by RNA polymerase III
*YDR011W (SNQ2)*	1.21	0.002116	Multidrug resistance Protein	Multidrug resistance
*YJL106W (IME2)*	1.21	0.067645	Meiosis induction protein kinase	Meiotic cell cycle; protein phosphorylation

### Validation of the RNA seq data by evaluation of a few genes by q‐RT‐PCR

To validate the accuracy of our RNA‐Seq data, we selected representative genes from different pathways for further analysis. Specifically, we examined three efflux pump genes (*PDR5*, *SNQ2*, *FEX2*) and the transcription factor encoding gene *SKN7*. All four genes showed upregulation in response to 5‐OP accumulation (Fig. S2).

### Evaluation of selected upregulated genes by either overexpression or deletion analysis

In the absence of any prior information about the targets of 5‐OP and the response to excess 5‐OP, we procured the deletions of a randomly selected list of upregulated genes from Euroscarf and examined them on plates/liquid medium containing higher levels of 5‐OP to examine whether any of the deletions showed enhanced sensitivity. This was carried out by comparing growth of these strains in liquid medium in the presence of 5‐oxoproline (5 mm) as well as on plate (100 mm). We deal with the different genes category wise.

### Efflux pumps of multiple pathways seem involved in the cellular response to 5‐OP

**Fig. 2:**
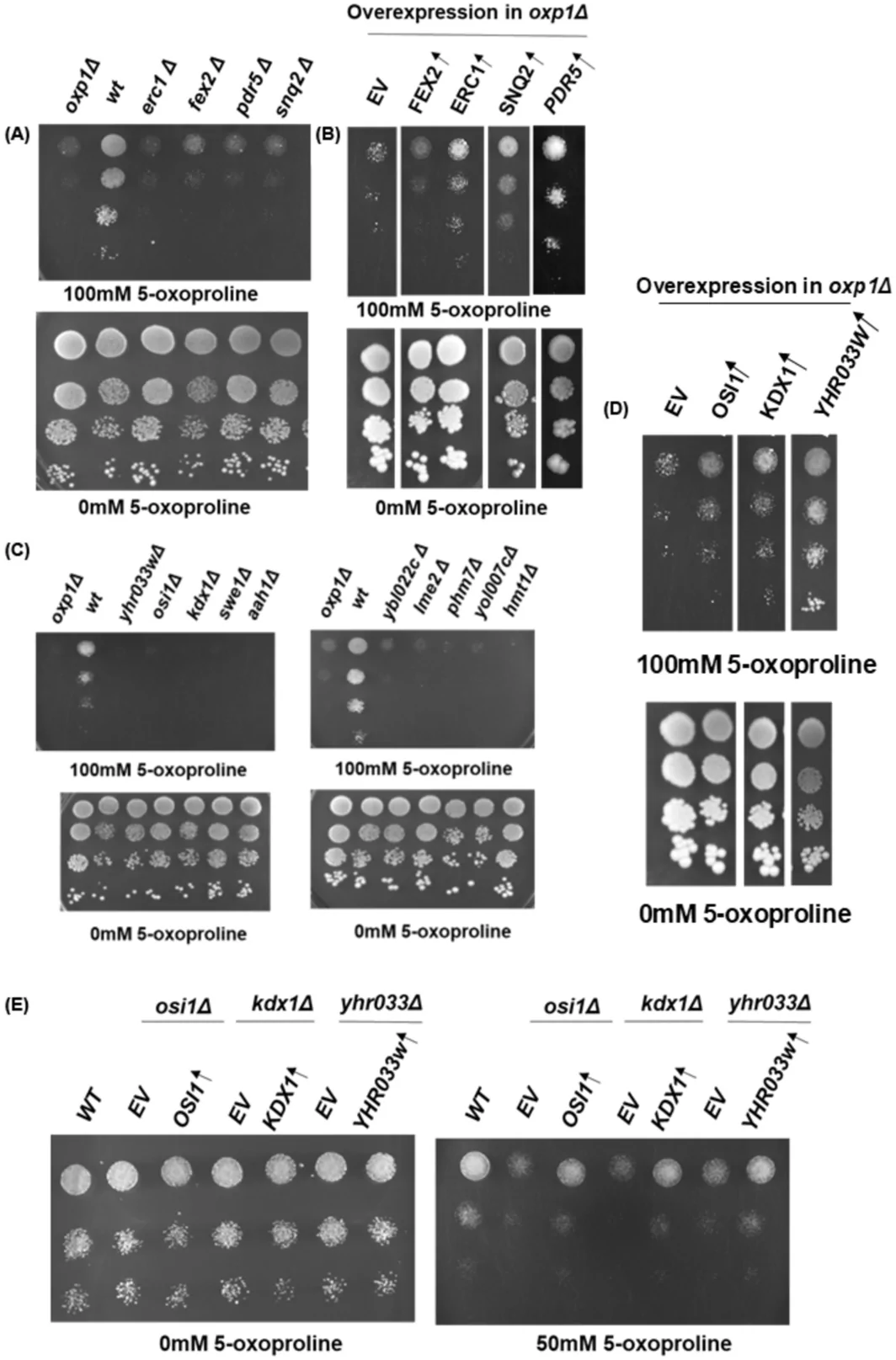
Involvement of efflux pumps and metabolic enzymes in 5‐oxoproline (5‐OP) resistance: (A) efflux deletion strains were sensitive to 5‐OP similar to *oxp1Δ*, compared to WT. (B) Overexpression of upregulated efflux pumps in *oxp1Δ* cells to evaluate resistance conferred on them. WT cells were BY4741 (AB5000), *oxp1Δ* was in BY4741 background. (C) Evaluation of different enzymes deletion strains on 5‐oxoproline, strains were compared with *oxp1Δ* and the WT (BY4741). (D) Overexpression of *OSI1*, *KDX1* and *YHR033W* genes in *oxp1Δ* cells confers resistance toward 5‐OP. Images were capture on day 4. (E) Overexpression of *OSI1*, *KDX1 and YHR033W* genes in their respective deletion strains confers resistance toward 5‐OP. All experiments were performed independently three times, data shown are representative images.

The RNA‐seq data revealed four efflux transporters that were upregulated. Interestingly, these four efflux proteins fell into three different types of transporters: the ABC transporters (Pdr5p, Snq2p), the multidrug/oligo‐saccharide‐lipid (polysaccharide) (MOP) exporter family (Erc1p), and the fluoride exporter (Fex) family. Pdr5p and Snq2p are known to represent functional and structural homologs of the mammalian MDR1 and MRP transporter [24]. Transcription of both *PDR5* and *SNQ2* in yeast is controlled by the transcription regulatory proteins Pdr1p and Pdr3p [25, 26]. The *ERC1* gene was primarily identified as a gene conferring ethionine resistance in *S. cerevisiae*, and overexpression of the *ERC1* in *S. cerevisiae* was earlier shown to lead to SAM accumulation [27]. Fex2p is involved in fluoride export and is part of a widespread family of conserved fluoride export proteins. *FEX2* is a paralog of *FEX1*, and deletion of both genes results in a large increase in fluoride sensitivity compared with the single mutant in yeast. It contains two FEX domains connected by a linker [28]. The upregulation of three of these efflux transporter genes (*PDR5*, *SNQ2*, *FEX2*) was validated through qRT‐PCR as described earlier (Fig. S2). The deletion of these strains was sensitive on plates containing 100 mm 5‐OP, suggesting their involvement in the efflux of 5‐OP (Fig. 2A). The growth was also confirmed in liquid media (Fig. S3). To further confirm their involvement in 5‐OP efflux, the genes were cloned downstream of the strong TEF promoter. The overexpression of *PDR5*, *SNQ2*, and *ERC1* genes was seen to provide resistance to high concentrations of 5‐OP on plates, which further confirms the involvement of these efflux pumps in rescuing the cell from the toxic effect of high amounts of 5‐OP in *oxp1Δ* cells (Fig. 2B). Only the overexpression of *FEX2* did not confer resistance, although we did not check if this was due to inadequate Fex2p expression. However, the deletion of *fex2Δ* was sensitive. Overexpression of *PDR5* rescued the defective phenotype of *pdr5Δ* (Fig. S4). Overall, this data suggests that these four efflux pumps of different families are all involved in the rescue of the cells from 5‐OP toxicity. These efflux pumps are known to export a wide range of compounds, and our data suggest that 5‐OP is also one of the substrates exported out by these pumps.

### Enzymes in different pathways appears to be targets of 5‐OP toxicity

A variety of different enzymes in different pathways were found to be upregulated during 5‐OP exposure. This also included a few potential enzymes of uncharacterized function. *YHR033W* encodes a putative glutamate 5‐kinase and a paralog of Pro1p protein, but no experimental evidence is present for its kinase activity. *OSI1* encodes a short‐chain dehydrogenase/reductase (SDR) protein with NADH‐dependent enzymatic activities for reduction of furfural (FF), glycolaldehyde (GA), formaldehyde (FA), and benzaldehyde (BZA) [29]. This protein was found to be upregulated under glycolaldehyde (GA) and furfural stress conditions (Wang *et al*., 2017). *KDX1* encodes a protein kinase that is implicated in the Slt2p mitogen‐activated (MAP) kinase signaling pathway. It is also known as a stress‐responsive protein and interacts with Rlm1p to activate *RCK1* gene expression in response to stress in *S. cerevisiae* [30]. As some of these could be potential targets of the accumulating 5‐OP, we evaluated deletions of these strains for any alterations in 5‐OP toxicity (Fig. 2C) and a few in liquid cultures (Fig. S3). The deletion strains for genes encoding these enzymes were sensitive to 5‐OP, while overexpression of *YHR033w*, *KDX1*, and *OSI1* confers resistance to 5‐OP toxicity in the *oxp1Δ* strain as well as their respective deletion strains (Fig. 2D,E). These data suggests that high levels of 5‐OP interfere with proper functioning of different metabolic pathways in the cell and disturb cellular homeostasis and result in defective growth.

### Investigating the role of different DNA binding proteins and transcription factors

**Fig. 3:**
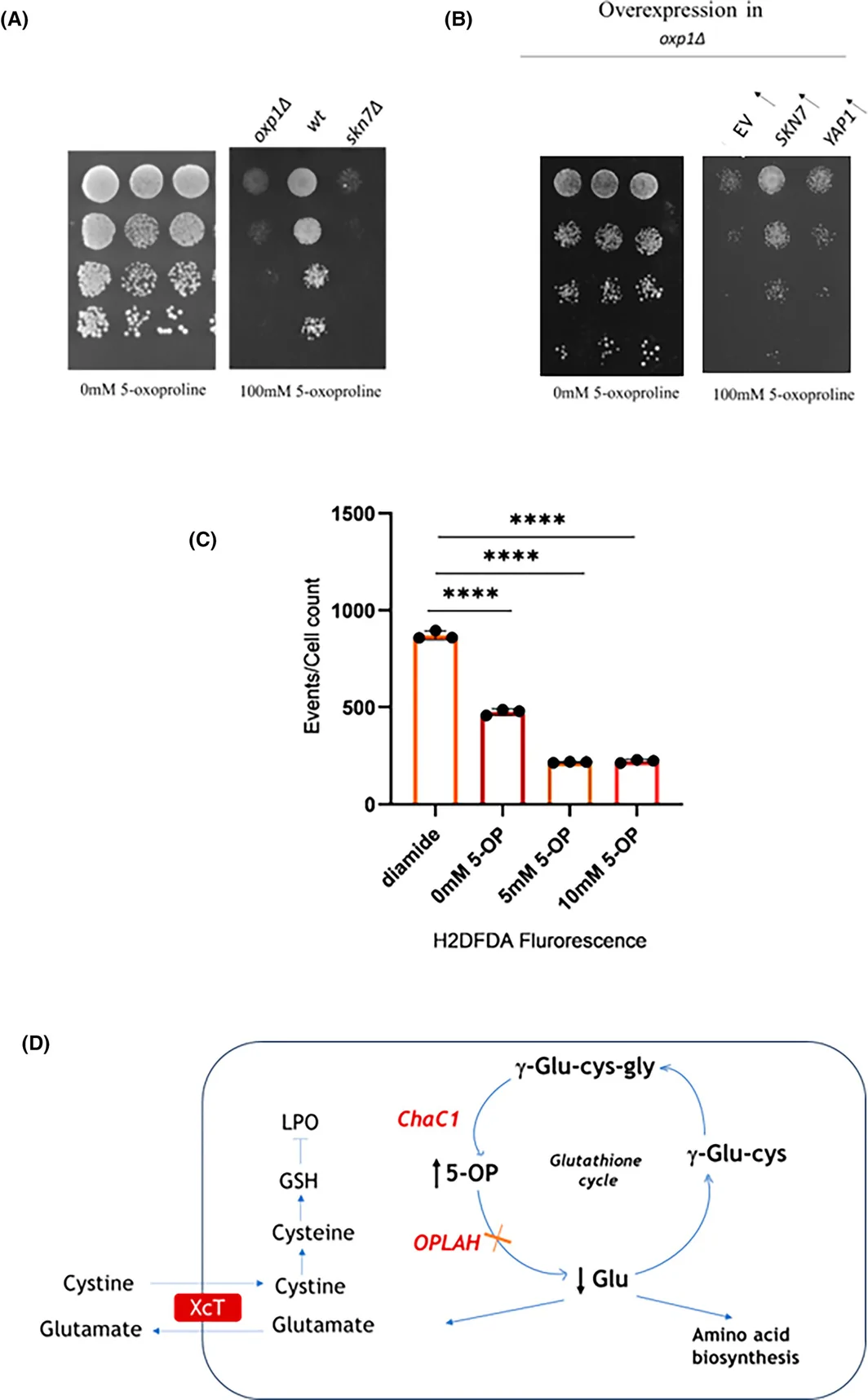
5‐oxoproline (5‐OP) sensitivity of oxidative stress response transcription factor deletions and ROS formation: (A) *skn7Δ* strain showed sensitivity to 100 mm 5‐OP compared with WT (BY4741). (B) Overexpression of *SKN7* conferred strong resistance to 5‐OP, whereas *YAP1* overexpression resulted in mild resistance. Images were capture by day 4. (C) ROS measurement by FACS using H2CDFDA dye: *oxp1Δ* cells were grown in minimal medium supplemented with varying concentration of 5‐OP, were incubated with H2CDFDA dye for 30 min; diamide treatment was used as a positive control. All experiments were performed in triplicates. Representative images are shown for the spotting assay. Statistical analysis was carried out using One‐way ANOVA with multiple comparisons. Significance is indicated as (****P < 0.0001), error bars represent SD. (D) Schematics showing an alternate hypothesis for 5‐OP mediated oxidative stress in mammalian system.

The transcriptomics data revealed genes encoding DNA binding proteins and transcription factors acting in diverse pathways to be upregulated. These include *SKN7*, *ELG1*, *RRT5*, *TBF1*, *SMP1*, and *HCM1*. We evaluated the deletion of the various transcription factors encoding genes. We observed that many of the transcription factor deletion strains showed a mild sensitivity to 5‐OP, but the maximum sensitivity was seen with *skn7Δ*. Since 5‐OP has been reported to cause oxidative stress and one of the stress response factors gene *SKN7* was upregulated, we examined this in greater detail. Its involvement was confirmed through the plate assay where the *skn7Δ* strain was sensitive to 5‐OP on the plate containing 100 mm 5‐OP (Fig. 3A). It was also evaluated through liquid culture (Fig. S3). Skn7p is a transcription factor, which works under stress conditions and also has a role in oxidative stress response in association with the Yap1p transcription factor [31]. Overexpression of *SKN7* in *oxp1Δ* strain provides resistance toward 5‐OP toxicity (Fig. 3B). However, overexpression of *YAP1* shows barely any resistance to 5‐OP compared to *SKN7 overexpression* (Fig. 3B). Other oxidative stress response transcription factor deletion strains such as *rrt5Δ*, *hcm1Δ*, *smp1Δ*, *msn2Δ*, and *msn4Δ* were not showing sensitivity toward 5‐OP, except *elg1Δ* and *tbf1Δ* showing mild sensitivity (Fig. S5).

### 5‐OP accumulation affects various membrane proteins in the cell

Among the list of upregulated genes, we have found the upregulation of a few membrane proteins upon 5‐OP exposure. This includes proteins with functions in cell wall integrity as well as some with unknown functions. In budding yeast, the cell wall integrity pathway is activated by several membrane proteins such as Wsc2p that act as sensors. These sensor proteins activate Pkc1, which in turn activates the MAPK module by phosphorylation [32]. The *S. cerevisiae* Flc2p protein is a putative calcium channel with a role in cell wall integrity; *FLC2* gene deletion resulted in pleiotropic phenotypes [33]. *PRM10* is selectively expressed during mating and encodes a multi‐spanning transmembrane protein. It has a role in plasma membrane fusion and localizes to sites of cell–cell contact where fusion occurs [34]. In our study, we observed that deletion of these genes shows sensitivity to 5‐OP on plate (Fig. S6), but the exact mechanisms would need further exploration.

### Evaluation of oxidative stress response in 5‐OP treated yeast cells

Since Skn7p, a stress response transcription factor, showed sensitivity to 5‐OP and 5‐OP was also suggested to cause oxidative stress in cardiac tissue, we decided to evaluate the oxidative stress. We evaluated the presence of ROS in the *oxp1Δ* cells grown in the presence of a varying concentration of 5‐OP using H2CDFDA dye. We did not observe any increase in ROS levels in the 5‐OP‐treated cells compared to diamide‐treated cells, which were used as a positive control (Fig. 3C). This result suggests that 5‐oxoproline accumulation does not cause ROS dependent oxidative stress.

### 5‐OP accumulation does not inhibit the Chac1 enzyme

The accumulation of 5‐OP induces a general stress response in cells, yet the initial steps triggering this response remain unclear, as 5‐OP itself appears to be an inert molecule. Further, in human cells, the 5‐OP accumulation was also accompanied by an oxidative stress response. The human Chac1 is an efficient degrader of glutathione, with one of the products being 5‐OP. Thus, there was a possibility that 5‐OP accumulation might be exhibiting feedback regulation of Chac1. We therefore investigated the effect of 5‐OP on the human Chac1 under *in vitro* conditions. We observed a very mild inhibition; this also was observed only at higher concentrations of 5‐OP (10 mm) (Fig. S7). Thus, it does not appear that Chac1 is a target of the elevated 5‐OP levels.

## Discussion

5‐oxoproline, as a metabolite, has been known for a long time. However, it has only been actively investigated as an N‐terminal moiety of proteins. As a metabolite in cells, there have been limited studies. Following the observations that 5‐oxoprolinase (OPLAH) knockdown has a higher propensity for heart failure in mouse and man has brought the substrate of OPLAH, 5‐oxoproline, into focus. To understand how higher 5‐OP in the cells affects cell metabolism, we needed a suitable model. The well‐defined yeast *S. cerevisiae* appeared to be an excellent eukaryotic model to investigate the consequences of 5‐OP. The ideal model of 5‐OP accumulation would have been one where the 5‐OP was generated endogenously. However, this approach using human Chac1 did not succeed in generating elevated levels of 5‐OP. It might be because the levels of GSH in the cell were not high enough. We eventually succeeded in creating a model that had between 12‐ and 20‐fold higher 5‐OP levels, substantially higher folds than observed in the mammalian system.

Investigations using this model revealed that elevated 5‐oxoproline is relatively well‐tolerated by cells. There was only a mild upregulation of genes of diverse pathways. The second key finding from this study is that 5‐OP does not cause oxidative stress. This is seen from both the transcriptomics studies and the ROS determination. However, the study did not test the changes in protein levels. Since 5‐OP is primarily formed from the degradation of GSH by Chac1, and since this has an indirect effect on the redox environment, we also checked whether Chac1 might be feedback regulated by one of its products (5‐OP). Glutathione concentrations in the cytoplasm are typically in the range of 1–2 mm (although in some cells such as hepatocytes it can be up to 10 mm) [35]. Concentrations of glutathione around 2 mm are also consistent with the Km of ChaC1 for glutathione (which is 2.2 mm) [36]. Given the concentrations of glutathione in the range of 2 mm, the concentrations of 5‐oxoproline (the product of the reaction with ChaC1) are unlikely to exceed 2 mm. However, with 2 mm 5‐oxoproline only 33% inhibition was observed. Even at 10 mm of 5‐oxoproline only 65% inhibition was observed. This limited inhibition of Chac1 cannot explain the severe downstream effects seen in cardiac cells even though it could be a partial contributor. Indeed, when we examine the cardiac study carefully, we see that the authors had found only a 2‐fold increase in plasma levels of 5‐oxoproline in OPLAH knockdown. This is not a significant increase because 5‐OP levels in plasma vary several folds only with dietary differences [14, 37]. One possible criticism of this study is that while what we are observing with yeast may not be true, in the case of mammalian cells the response could be different and one might be experiencing oxidative stress response in the mammalian system, though not the yeast model. However, the observations are consistent with the earlier suggested role of 5‐oxoproline as relatively inert with a role as an osmoprotectant.

If 5‐oxoproline (5‐OP) levels are not the direct cause of the observed oxidative stress and phenotypic consequences, then what might be the possible explanation? One plausible alternative is that the downstream metabolite of 5‐OP, glutamate, produced by the enzyme 5‐oxoprolinase (OPLAH), plays a critical role. Glutamate could contribute to maintaining redox balance and cellular homeostasis by enhancing cysteine availability through the cystine/glutamate antiporter (XcT). This exchange mechanism imports cystine in exchange for glutamate, and once inside the cell, cystine is reduced to cysteine, a rate‐limiting precursor for glutathione (GSH) synthesis. Therefore, disruptions in 5‐OP metabolism could indirectly affect glutathione biosynthesis and redox buffering capacity by altering intracellular glutamate and cysteine levels. Additionally, glutamate serves as a key amino acid in multiple metabolic pathways, and its altered availability might influence cellular energy metabolism, amino acid balance, or signaling processes that collectively contribute to the observed phenotypes. Importantly, since both glutamate export and cysteine import via system XcT are tightly linked to ferroptosis regulation, changes in glutamate or cysteine availability resulting from altered 5‐OP metabolism could significantly impact cellular susceptibility to oxidative stress (Fig. 3D). More studies would be needed to confirm the validity of this possibility. However, as yeast lack XcT, it would have to be evaluated in a mammalian model.

## Conflict of interest

There is no conflict of interest for any of the authors.

## Author contributions

PD performed most of the experiments, data analysis, and manuscript writing; VBS performed MCT‐1 model experiments, data analysis; PS performed 5‐OP quantification and data analysis; SSG supervised the design and analysis of the 5‐OP quantification experiments; AKB was involved in supervision, experiment designing, conceptualization, and manuscript writing.

## Supporting information


**Fig. S1.** (A) Growth curve analysis comparing human Chac1‐overexpressing cells carrying either empty vector or co‐expressing TEF‐OXP1. Only a mild growth difference was observed.
**Fig. S2.** Validation of RNA‐seq–identified upregulated genes in *oxp1Δ* cells using qRT‐PCR.
**Fig. S3.** Growth analysis of deletion strains in liquid media. All the experiments were done in triplicates, for *pdr5Δ*, *skn7Δ* and *snq2Δ*, *a*ll three datapoints are shown, whereas for *erc1Δ*, *kdx1Δ*, *yhr033wΔ*, *msn4Δ* and *msn2Δ*, a representative is shown.
**Fig. S4.** PDR5 overexpression show resistance toward 5‐OP in *pdr5Δ* strain, this experiment was repeated to confirm and this is the representative picture. Images were capture by day 4.
**Fig. S5.** Deletion of other transcription factors did not show 5‐OP sensitivity: Evaluation of transcription factor deletion strains on 5‐OP plates compared with WT (BY4741).
**Fig. S6.** Evaluation of different membrane protein deletion strains on 5‐oxoproline, strains were compared with *oxp1Δ* and the WT (BY4741).
**Fig. S7.** *In vitro* inhibition of recombinant human Chac1 by increasing concentrations of 5‐OP, with activity measured as described in Materials and Methods.
**Table S1.** List of strains used in the study.
**Table S2.** List of primers used in the study.
**Table S3.** Gene ontology analysis of RNA seq data, top biological processes selected using the *e* value <0.05 and fold enrichment >10.
**Table S4.** List of downregulated genes.

## Data Availability

All data are contained within the manuscript. RNA sequencing data was deposited in NCBI (Accession no. PRJNA1345586).
