# Supplementary material for: A yeast model of 5‐oxoproline accumulation reveals a general toleration to 5‐oxoproline
Source: FEBS Open Bio. 2026 Jul 15:10.1002/2211-5463.70254. Online ahead of print. doi: 10.1002/2211-5463.70254 (PMC13398684; doi:10.1002/2211-5463.70254)
Supplement: Supplementary file 1 — Fig. S1. (A) Growth curve analysis comparing human Chac1‐overexpressing cells carrying either empty vector or co‐expressing TEF‐OXP1. Only a mild growth difference was observed. Fig. S2. Validation of RNA‐seq–identified upregulated genes in oxp1Δ cells using qRT‐PCR. Fig. S3. Growth analysis of deletion strains in liquid media. All the experiments were done in triplicates, for pdr5Δ, skn7Δ and snq2Δ, all three datapoints are shown, whereas for erc1Δ, kdx1Δ, yhr033wΔ, msn4Δ and msn2Δ, a representative is shown. Fig. S4. PDR5 overexpression show resistance toward 5‐OP in pdr5Δ strain, this experiment was repeated to confirm and this is the representative picture. Images were capture by day 4. Fig. S5. Deletion of other transcription factors did not show 5‐OP sensitivity: Evaluation of transcription factor deletion strains on 5‐OP plates compared with WT (BY4741). Fig. S6. Evaluation of different membrane protein deletion strains on 5‐oxoproline, strains were compared with oxp1Δ and the WT (BY4741). Fig. S7. In vitro inhibition of recombinant human Chac1 by increasing concentrations of 5‐OP, with activity measured as described in Materials and Methods. Table S1. List of strains used in the study. Table S2. List of primers used in the study. Table S3. Gene ontology analysis of RNA seq data, top biological processes selected using the e value <0.05 and fold enrichment >10. Table S4. List of downregulated genes. [file FEB4-9999-0-s001.docx]

**Yeast model reveals a general toleration to 5-oxoproline accumulation**


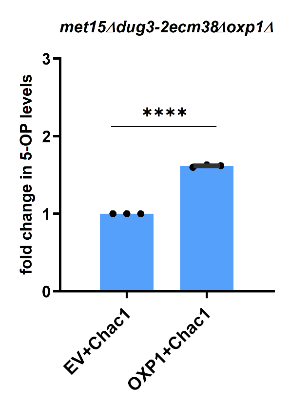
Supplementary data:


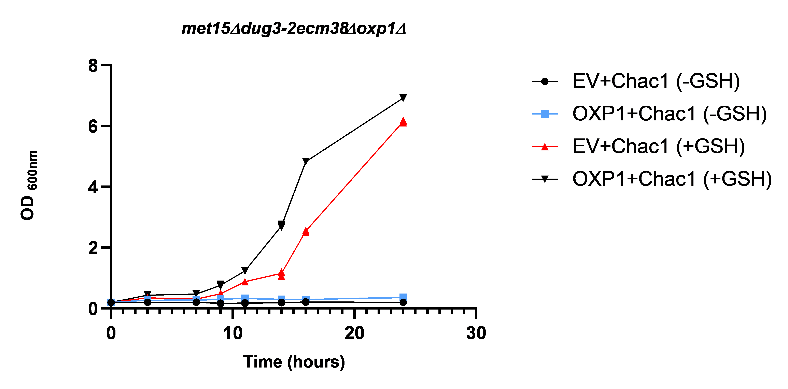


B

A


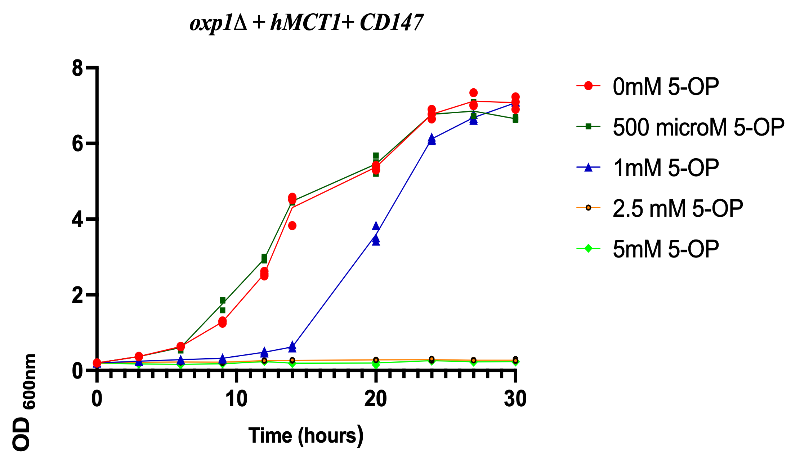

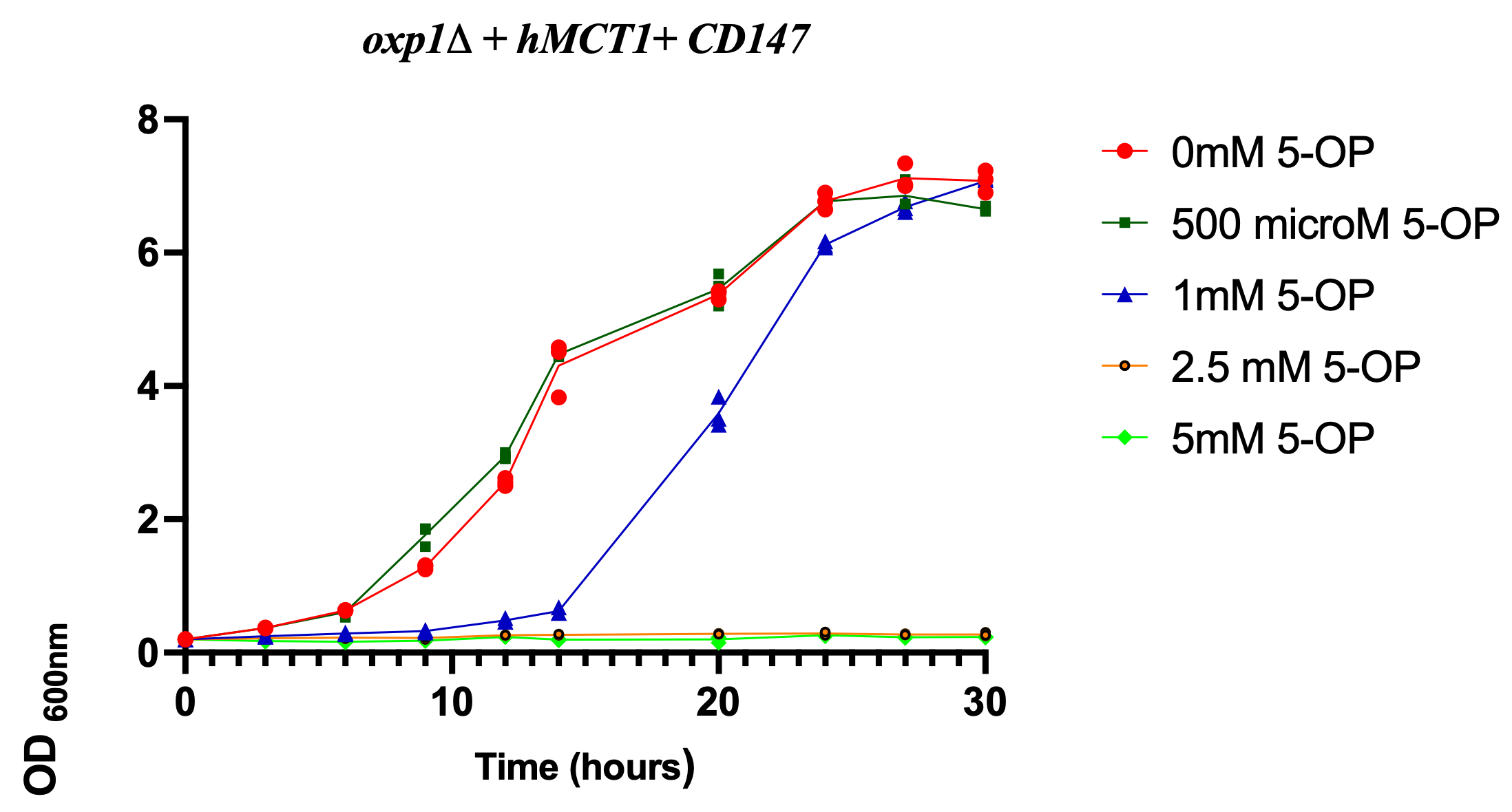


C

D


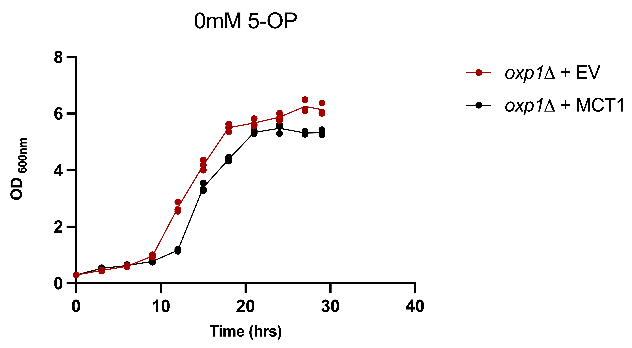

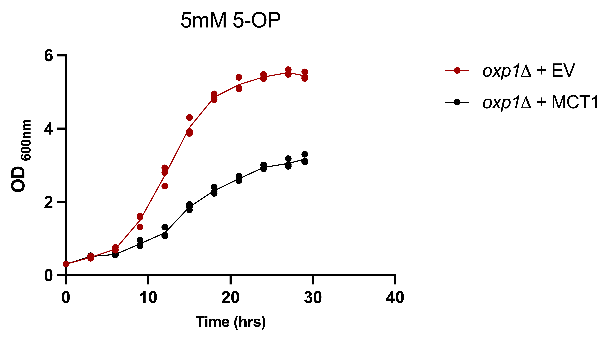


E


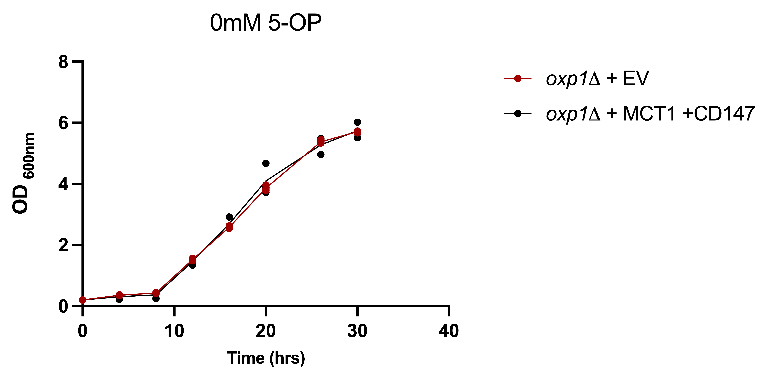

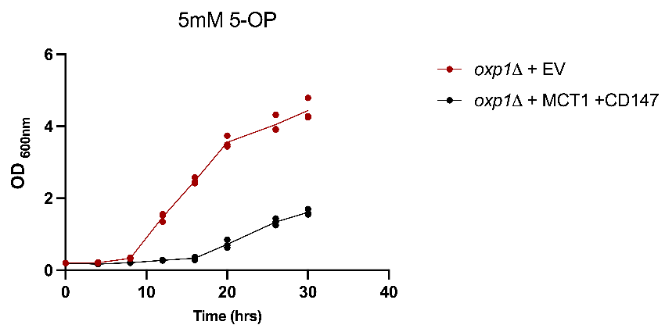


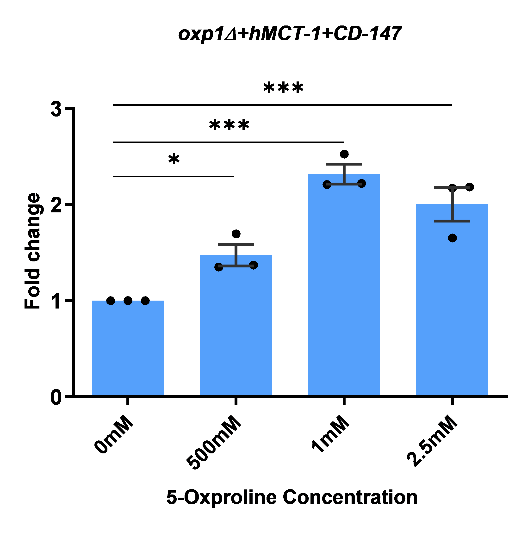


F

G


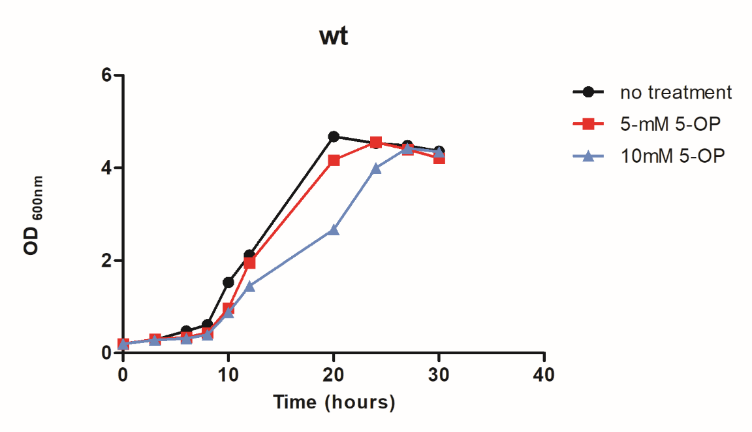


Fig S1: A) Growth curve analysis comparing human Chac1-overexpressing cells carrying either empty vector or co-expressing TEF-OXP1. Only a mild growth difference was observed. B) Quantification of 5-oxoproline (5-OP) in Chac1-overexpressing cells co-expressing either OXP1 or empty vector. Samples were collected and 5-OP estimated as described in Materials and Methods. C) Growth analysis of *oxp1Δ* cells transformed with MCT-1 and CD-147 on lower concentrations of 5-OP (500 μM, 1 mM, and 2.5 mM). Cells grown on 1 mM 5-OP showed a significantly defective growth phenotype and were selected for further studies. D) growth pattern of *oxp1Δ* cells with only MCT-1 overexpression as well as E) along with CD-147, *oxp1Δ* cells with MCT-1 shows severe growth defect on 5mM 5-OP whereas MCT-1 with CD-147 transformed *oxp1Δ* cells were not able to grow. F) Mass spectrometry-based quantification of 5-OP in MCT-1/CD-147-transformed *oxp1Δ* cells exposed to 1 mM and 2.5 mM 5-OP, showing significantly elevated 5-OP levels. G) Growth pattern of WT (BY4741) cells grown on SD media containing 5mM 5-OP and 10mM 5-OP. All experiments were performed independently three times. Statistical analysis was carried out using Student’s t-test (B) and one way ANOVA with multiple comparisons (F) Significance is indicated as (*p < 0.05, ***p < 0.001, ****p < 0.0001). error bars represent SEM.


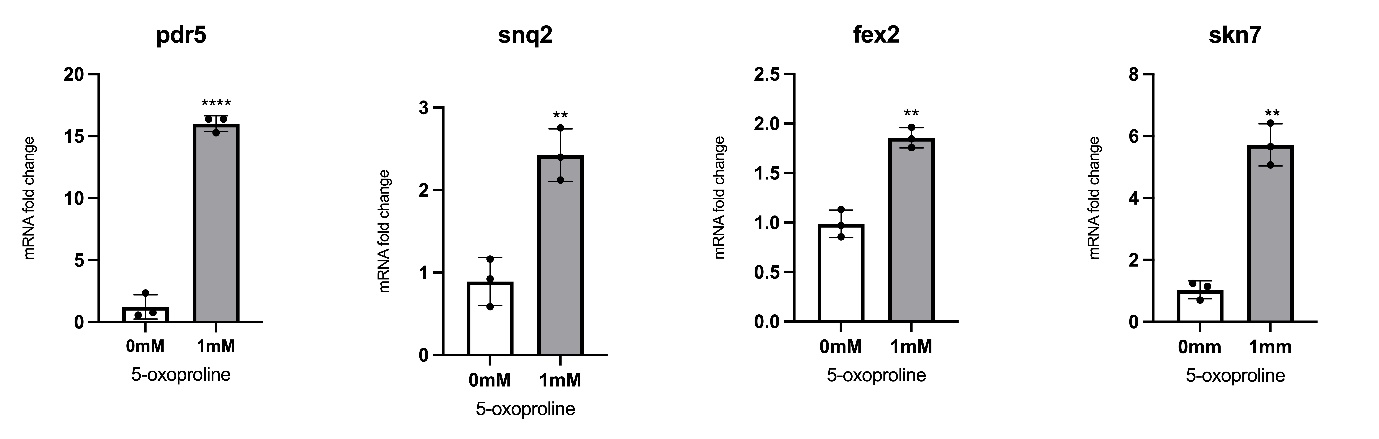


Fig S2: Validation of RNA-seq–identified upregulated genes in *oxp1Δ* cells using qRT-PCR. All experiments were performed independently three times. Statistical analysis was carried out using Student’s t-test. Significance is indicated as (*p < 0.01, **p < 0.001). error bars indicate SD.


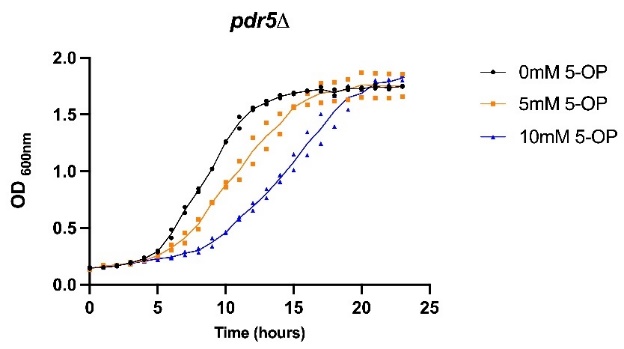

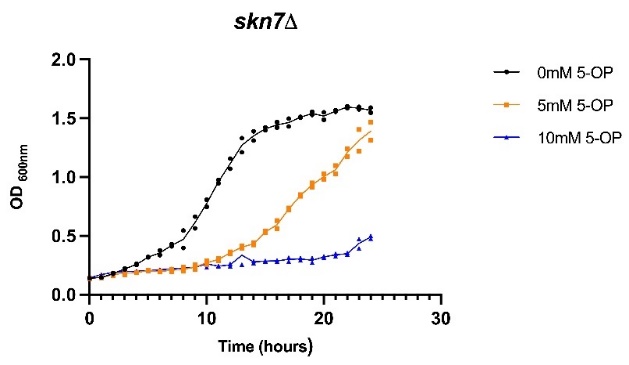

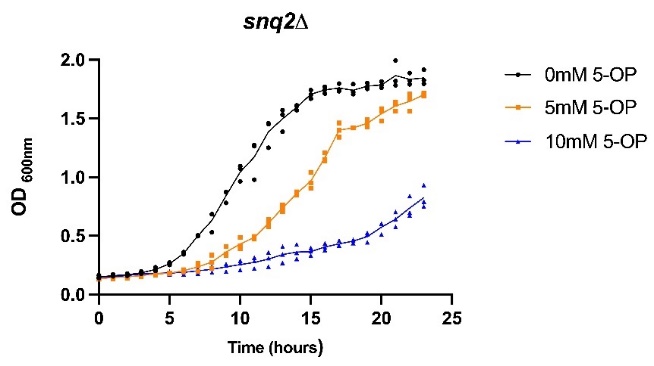

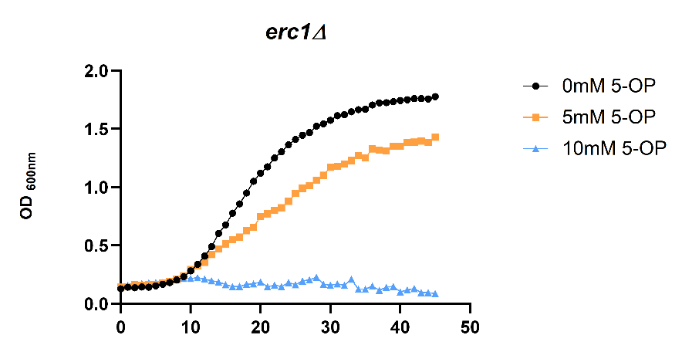


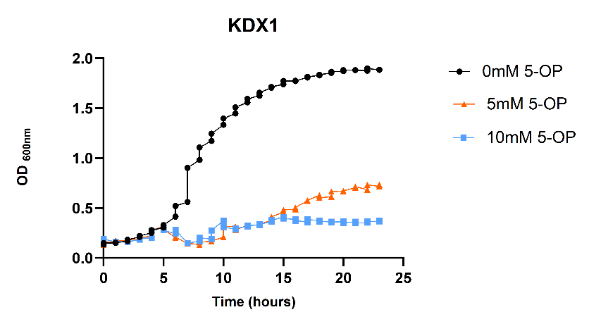

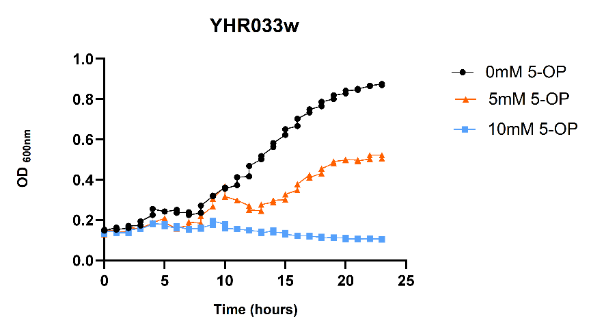

Fig S3: growth analysis of deletion strains in liquid media. All the experiments were done in triplicates, for *pdr5Δ*, *skn7Δ* and *snq2Δ , a*ll three datapoints are shown whereas for *erc1Δ*, *kdx1Δ*, *yhr033wΔ*, *msn4Δ* and *msn2Δ*, a representative is shown.


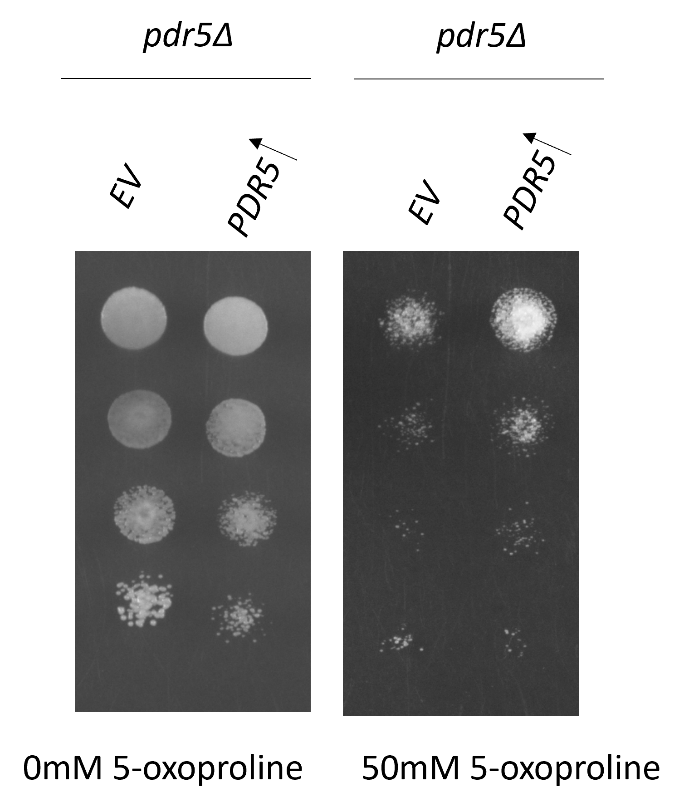


Fig S4: PDR5 overexpression show resistance towards 5-OP in *pdr5Δ* strain, this experiment was repeated to confirm and this is the representative picture. Images were capture by day 4.


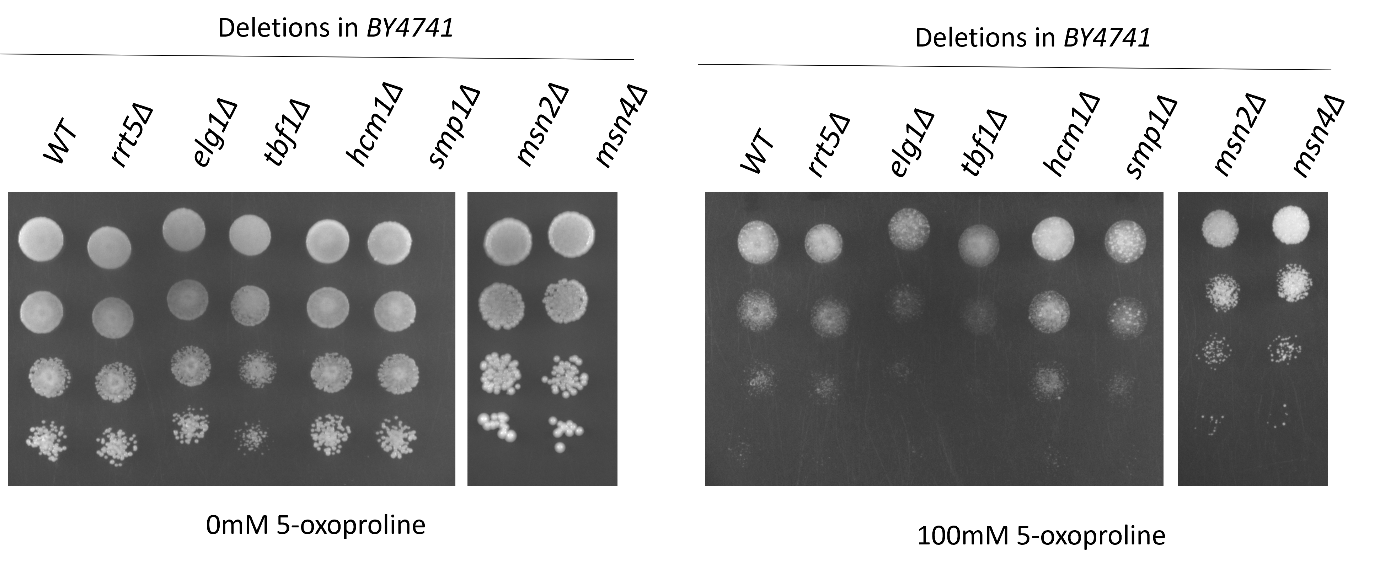


Fig S5: Deletion of other transcription factors did not show 5-OP sensitivity: Evaluation of transcription factor deletion strains on 5-OP plates compared with WT (BY4741). This experiment was repeated to confirm and this is the representative picture Images were capture by day 4.


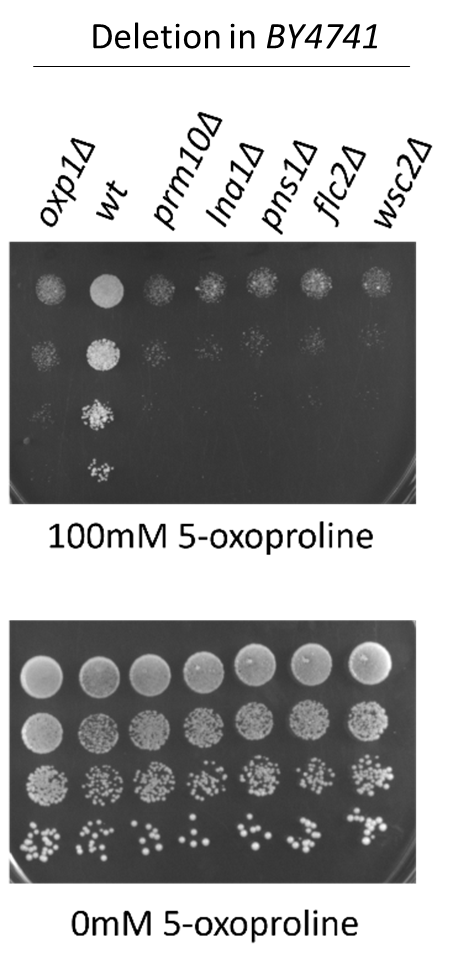


Fig S6: Evaluation of different membrane protein deletion strains on 5-oxoproline, strains were compared with *oxp1Δ* and the WT (BY4741). The deletions strains were sensitive for 5-OP, this experiment was repeated three time and a representative data is presented. Images were capture by day 4.


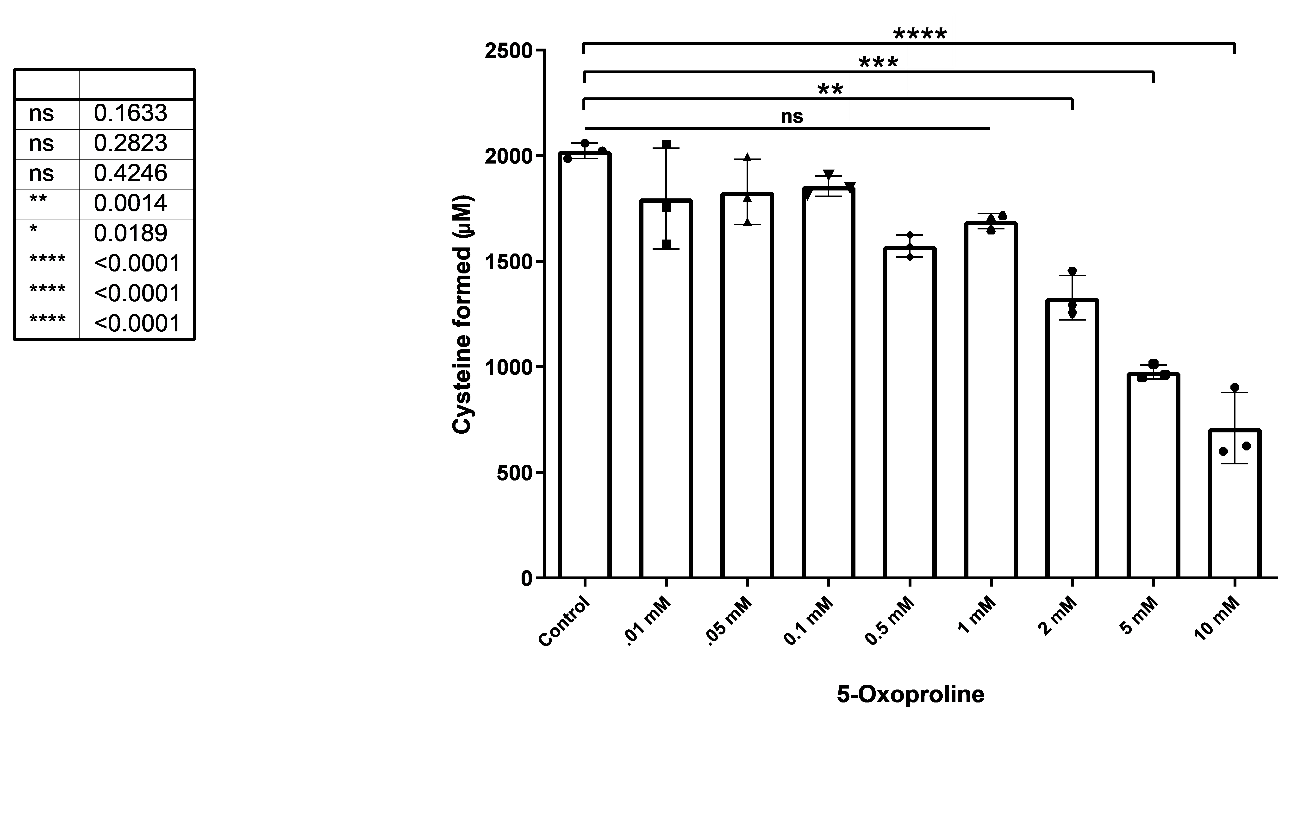


Fig S7: In vitro inhibition of recombinant human Chac1 by increasing concentrations of 5-OP, with activity measured as described in Materials and Methods. Statistical analysis was carried out using One-way ANOVA with multiple comparisons. Significance is indicated as (ns- non significant, **p < 0.01, ***p < 0.001, ****p < 0.0001), error bars represent SD.

Supplementary Table1: List of strains used in the study

| Strain | Genotype | Source |
| --- | --- | --- |
| AB460 | *E. coli* DH5α | Lab stock |
| AB5000 | *BY4741MATahis3Δ1leu2Δ0met15Δ0ura3Δ0* | Lab stock |
| AB6302 | *BY4741MATa his3Δ1leu2Δ 0met15Δ0ura3Δ0oxp1Δ::KanMX* | Lab stock |
| AB1723 | *MATαhis3∆1leu2∆0lys2∆0met15∆0ura3∆0ecm38∆::KanMX4dug3-2* | Lab stock |
| AB6486 | *MATαhis3∆1leu2∆0 met15∆0 ura3∆0 dug3∆oxp1∆::LEU2* | Lab stock |
| AB6547 | *MATαhis3∆1leu2∆0lys2∆0met15∆0ura3∆0ecm38∆::KanMX4 dug3-2oxp1∆:: LEU2* | Lab stock |
| AB6259 | *BY4741MATahis3Δ1leu2Δ0met15Δ0ura3Δ0pdr5Δ::KanMX* | Euroscarf |
| AB681 | *BY4741MATahis3Δ1leu2Δ0met15Δ0ura3Δ0snq2Δ::KanMX* | Euroscarf |
| AB6642 | *BY4741MATahis3Δ1leu2Δ0met15Δ0ura3Δ0erc1Δ::KanMX* | Euroscarf |
| AB6634 | *BY4741MATahis3Δ1leu2Δ0met15Δ0ura3Δ0osi1Δ::KanMX* | Euroscarf |
| AB6618 | *BY4741MATahis3Δ1leu2Δ0met15Δ0ura3Δ0 yhr033wΔ::KanMX* | Euroscarf |
| AB6620 | *BY4741MATahis3Δ1leu2Δ0met15Δ0ura3Δ0swe1Δ::KanMX* | Euroscarf |
| AB6628 | *BY4741MATahis3Δ1leu2Δ0met15Δ 0ura3Δ0aah1Δ::KanMX* | Euroscarf |
| AB6629 | *BY4741MATahis3Δ1leu2Δ0met15Δ0ura3Δ0ybl022cΔ::KanMX* | Euroscarf |
| AB6623 | *BY4741MATahis3Δ1leu2Δ0met15Δ0ura3Δ0ime2Δ::KanMX* | Euroscarf |
| AB6625 | *BY4741MATahis3Δ1leu2Δ0met15Δ0ura3Δ0phm7Δ::KanMX* | Euroscarf |
| AB6624 | *BY4741MATahis3Δ1leu2Δ0met15Δ0ura3Δ 0yol007cΔ::KanMX* | Euroscarf |
| AB6630 | *BY4741MATahis3Δ1leu2Δ0met15Δ0ura3Δ0hmt1Δ::KanMX* | Euroscarf |
| AB1032 | *BY4741MATahis3Δ1leu2Δ0met15Δ0ura3Δ0skn7Δ::KanMX* | Euroscarf |
| AB1034 | *BY4741MATahis3Δ1leu2Δ0met15Δ0ura3Δ0yap1Δ::KanMX* | Euroscarf |
| AB6621 | *BY4741MATahis3Δ1leu2Δ0met15Δ0ura3Δ0prm10Δ::KanMX* | Euroscarf |
| AB6636 | *BY4741MATahis3Δ1leu2Δ0met15Δ0ura3Δ0ina1Δ::KanMX* | Euroscarf |
| AB6627 | *BY4741MATahis3Δ1leu2Δ0met15Δ0ura3Δ0pns1Δ::KanMX* | Euroscarf |
| AB6617 | *BY4741MATahis3Δ1leu2Δ0met15Δ0ura3Δ0flc2Δ::KanMX* | Euroscarf |
| AB6619 | *BY4741MATahis3Δ1leu2Δ0met15Δ0ura3Δ0wsc2Δ::KanMX* | Euroscarf |

Supplementary Table 2: List of primers used in the study

| Primer Name | SEQUENCE (5’-3’) |
| --- | --- |
| RP_416CEN | CTTCTGTTCGGAGATTACCGAATC |
| FP_416CEN | TCTAGAAAACTTAGATTAGATTGC |
| FP_416Ura | TCGCGCGTTTCGGTGATGAC |
| RP_416Ura | CTCGAGTCATGTAATTAGTTATGT |
| Oxp1 del check FP | CAGTAATAATTTTGAGCATTGA |
| Oxp1 del check RP | TGGTTCCGTGTAATTCGATATT |
| FP-hSLC16A1-BamHI | GACCAAGGATCCATGCCACCAGCAGTTGGAG |
| RP-hSLC16A1-XhoI | GATTACCTCGAGTCAGACTGGACTTTCCTC |
| FP CD147-EcoR-I | GATCATGAATTCATGGCGGCTGCGCTGTTC |
| RP CD147-Xho-I | CATTAGCTCGAGTCAGGAAGAGTTCCTCTGG |
| Oxp1 leu FP | ATGCAGAAAGGAAACATAAGAATTGCCATCGATAAGGGTGAACTGTGGGAATACTCAGGT |
| Oxp1 leu RP | ATGCAGAAAGGAAACATAAGAATTGCCATCGATAAGGGTGAACTGTGGGAATACTCAGGT |
| PDR5 FP-RT | CAGTTGCATGAAAGGGGTGC |
| PDR5 RP-RT | CGTTAGCAACACCAACAGCC |
| SKN7 FP-RT | TAAAACCACCGGCGATAGCA |
| SKN7 RP-RT | TCGTGTTCATCAGTCGAGGC |
| SNQ2 FP-RT | CTTCATCCACTTCCGGTGCT |
| SNQ2 RP-RT | AAAGATGCCAGAGTGGAGCC |
| YAP1 FP-RT | TTCCTCCAGCGCTACTTTGG |
| YAP1 RP-RT | AGCCGAGATGGGTTTCTTGG |
| ERC1 FP-RT | TTGAGGGCGAAGAGCAAGAG |
| ERC1 RP-RT | TGTTGCCACGCTTCTTGTTG |
| FEX2 FP-RT | GTGAACTCGTTGAATTCATGCC |
| FEX2 RP-RT | ACTCAAAGTGCCGCAAAATCC |
| KDX1-FP | TTAGAAAGAAAGCATAGCAATCTAATCTAAGTTTTCTAGAGGATCCATGGCGACTGACAC |
| KDX1-RP | GGAGGGCGTGAATGTAAGCGTGACATAACTAATTACATGACTCGAGTTAGTTAACACCCTG |
| OSI-FP | TTAGAAAGAAAGCATAGCAATCTAATCTAAGTTTTCTAGAGGATCCATGAATACTTCATC |
| OSI-RP | GGAGGGCGTGAATGTAAGCGTGACATAACTAATTACATGACTCGAGCTAAAAGACGCCTTC |
| YHR033W-FP | TTAGAAAGAAAGCATAGCAATCTAATCTAAGTTTTCTAGAGGATCCATGACAAAAGCTTA |
| YHR033W-RP | GGAGGGCGTGAATGTAAGCGTGACATAACTAATTACATGACTCGAGTCAAAATTGCGGAG |
| ERC1-FP | TTAGAAAGAAAGCATAGCAATCTAATCTAAGTTTTCTAGAGGATCCATGTCTAAACAATT |
| ERC1-RP | GAGGGCGTGAATGTAAGCGTGACATAACTAATTACATGACTCGAGCTAGTTATACCCAAC |
| FEX2-FP | TTAGAAAGAAAGCATAGCAATCTAATCTAAGTTTTCTAGAGGATCCATGATTTTCAATCC |
| FEX2-RP | GGAGGGCGTGAATGTAAGCGTGACATAACTAATTACATGACTCGAGCTAACAAATCGGGT |

Supplementary Table 3: Gene ontology analysis of RNA seq data, top biological processes selcectd using the *e value<0.05* and fold enrichment >10

Upregulated biological process

| **Enrichment FDR** | **nGenes** | **Pathway Genes** | **Fold Enrichment** | **Pathways (click for details)** |
| --- | --- | --- | --- | --- |
| 4.2E-02 | 3 | 9 | 21.8 | [Xenobiotic export from cell](https://amigo.geneontology.org/amigo/term/GO:0046618) |
| 4.2E-02 | 3 | 9 | 21.8 | [Xenobiotic detoxification by transmembrane export across the plasma membrane](https://amigo.geneontology.org/amigo/term/GO:1990961) |
| 4.4E-03 | 5 | 17 | 19.2 | [Export across plasma membrane](https://amigo.geneontology.org/amigo/term/GO:0140115) |
| 3.4E-02 | 8 | 100 | 5.2 | [Rna methylation](https://amigo.geneontology.org/amigo/term/GO:0001510) |
| 3.4E-02 | 9 | 126 | 4.7 | [Macromolecule methylation](https://amigo.geneontology.org/amigo/term/GO:0043414) |
| 3.4E-02 | 10 | 152 | 4.3 | [Methylation](https://amigo.geneontology.org/amigo/term/GO:0032259) |
| 3.4E-02 | 19 | 498 | 2.5 | [Transmembrane transport](https://amigo.geneontology.org/amigo/term/GO:0055085) |

Downregulated biological process

| **Enrichment FDR** | **nGenes** | **Pathway Genes** | **Fold Enrichment** | **Pathways (click for details)** |
| --- | --- | --- | --- | --- |
| 1.3E-04 | 10 | 18 | 5.8 | [Mitochondrial electron transport cytochrome c to oxygen](https://amigo.geneontology.org/amigo/term/GO:0006123) |
| 5.1E-12 | 27 | 52 | 5.4 | [Mitochondrial respiratory chain complex assembly](https://amigo.geneontology.org/amigo/term/GO:0033108) |
| 1.7E-12 | 29 | 56 | 5.4 | [Mitochondrial respirasome assembly](https://amigo.geneontology.org/amigo/term/GO:0097250) |
| 1.3E-04 | 11 | 22 | 5.2 | [Atp biosynthetic proc.](https://amigo.geneontology.org/amigo/term/GO:0006754) |
| 1.3E-04 | 11 | 22 | 5.2 | [Proton motive force-driven atp synthesis](https://amigo.geneontology.org/amigo/term/GO:0015986) |
| 9.7E-06 | 15 | 32 | 4.9 | [Respiratory chain complex iv assembly](https://amigo.geneontology.org/amigo/term/GO:0008535) |
| 9.7E-06 | 15 | 32 | 4.9 | [Mitochondrial cytochrome c oxidase assembly](https://amigo.geneontology.org/amigo/term/GO:0033617) |
| 1.7E-06 | 19 | 44 | 4.5 | [Cytochrome complex assembly](https://amigo.geneontology.org/amigo/term/GO:0017004) |
| 8.9E-06 | 18 | 44 | 4.3 | [Atp synthesis coupled electron transport](https://amigo.geneontology.org/amigo/term/GO:0042773) |
| 8.9E-06 | 18 | 44 | 4.3 | [Mitochondrial atp synthesis coupled electron transport](https://amigo.geneontology.org/amigo/term/GO:0042775) |
| 2.2E-05 | 17 | 43 | 4.1 | [Aerobic electron transport chain](https://amigo.geneontology.org/amigo/term/GO:0019646) |
| 9.8E-06 | 19 | 50 | 4 | [Oxidative phosphorylation](https://amigo.geneontology.org/amigo/term/GO:0006119) |
| 2.9E-05 | 18 | 49 | 3.8 | [Respiratory electron transport chain](https://amigo.geneontology.org/amigo/term/GO:0022904) |
| 3.9E-05 | 18 | 50 | 3.8 | [Electron transport chain](https://amigo.geneontology.org/amigo/term/GO:0022900) |
| 2.9E-05 | 21 | 64 | 3.4 | [Mitochondrial membrane organization](https://amigo.geneontology.org/amigo/term/GO:0007006) |
| 1.6E-07 | 39 | 140 | 2.9 | [Mitochondrial translation](https://amigo.geneontology.org/amigo/term/GO:0032543) |
| 2.1E-12 | 63 | 230 | 2.9 | [Mitochondrion organization](https://amigo.geneontology.org/amigo/term/GO:0007005) |
| 5.7E-05 | 31 | 126 | 2.6 | [Cellular respiration](https://amigo.geneontology.org/amigo/term/GO:0045333) |
| 2.2E-05 | 39 | 171 | 2.4 | [Mitochondrial gene expression](https://amigo.geneontology.org/amigo/term/GO:0140053) |
| 9.2E-06 | 103 | 646 | 1.7 | [Protein-containing complex assembly](https://amigo.geneontology.org/amigo/term/GO:0065003) |

Supplementary Table S4: list of downregulated genes

| **Gene_Name_GFF** | **log2FoldChange** | **Pvalue** | **Protein Names** | **Function** |
| --- | --- | --- | --- | --- |
| **ZPS1** | -4.99808927 | 1.00877E-28 | Protein ZPS1 | Putative zinc-binding cell-wall/vacuolar protein; exact function unknown |
| **SPG1** | -3.619288043 | 5.45633E-07 | Stationary phase gene 1 protein | Stationary-phase associated protein; exact role unknown |
| **GPM2** | -1.561548949 | 7.52752E-07 | Phosphoglycerate mutase 2 | Putative phosphoglycerate mutase-like protein; function not well defined |
| **YBR285W (HAB1)** | -2.491256004 | 7.64701E-07 | YBR285W isoform 1 | Autophagy-related protein; binds ribosome; involved in autophagosome formation |
| **ASP3-1** | -2.915763568 | 1.15081E-06 | L-asparaginase II | Hydrolyzes L-asparagine → L-aspartate + NH₃; induced under nitrogen starvation |
| **CYC7** | -3.242196569 | 1.56532E-06 | Cytochrome c isoform 2 | Electron carrier in mitochondrial electron transport chain |
| **YGR174W-A** | -3.309987915 | 3.99782E-06 | Iso-2 cytochrome c | Likely a cytochrome c–related protein; function not well defined |
| **SPG4** | -3.397099725 | 5.92546E-06 | Stationary phase protein 4 | Stationary-phase induced protein; function unknown |
| **EGO4** | -3.505856702 | 6.55662E-06 | Exit from rapamycin-induced growth arrest protein | Member of EGO/GSE complex; regulates microautophagy and TORC1 signaling |
| **SUE1** | -2.242726368 | 1.58347E-05 | Protein SUE1, mitochondrial | Mitochondrial protein involved in protein quality control / protein degradation |
| **UIP4** | -1.573174773 | 1.75392E-05 | ULP1-interacting protein 4 | Unknown; interacts with SUMO protease ULP1; likely involved in SUMO pathway |
| **YAP6** | -2.502987592 | 1.87499E-05 | Transcription factor YAP6 | Transcriptional repressor involved in osmotic & stress-related gene regulation |
